# Composition Profiling and Authenticity Assessment of Camellia Oil Using High Field and Low Field ^1^H NMR

**DOI:** 10.3390/molecules26164738

**Published:** 2021-08-05

**Authors:** Meijun Xing, Shenghao Wang, Jianzhong Lin, Feng Xia, Jianghua Feng, Guiping Shen

**Affiliations:** 1 Fujian Provincial Key Laboratory of Plasma and Magnetic Resonance, Department of Electronic Science, Xiamen University, Xiamen 361005, China; 33320201150302@stu.xmu.edu.cn (M.X.); shenghaowang2017@163.com (S.W.); xiafeng@xmu.edu.cn (F.X.); jianghua.feng@xmu.edu.cn (J.F.); 2 Technology Center of Xiamen Customs, Xiamen 361012, China; jiangzhong2021@126.com

**Keywords:** food quality, nuclear magnetic resonance, pattern recognition, authenticity, camellia oil

## Abstract

Camellia oil (CA), mainly produced in southern China, has always been called Oriental olive oil (OL) due to its similar physicochemical properties to OL. The high nutritional value and high selling price of CA make mixing it with other low-quality oils prevalent, in order to make huge profits. In this paper, the transverse relaxation time (T_2_) distribution of different brands of CA and OL, and the variation in transverse relaxation parameters when adulterated with corn oil (CO), were assessed via low field nuclear magnetic resonance (LF-NMR) imagery. The nutritional compositions of CA and OL and their quality indices were obtained via high field NMR (HF-NMR) spectroscopy. The results show that the fatty acid evaluation indices values, including for squalene, oleic acid, linolenic acid and iodine, were higher in CA than in OL, indicating the nutritional value of CA. The adulterated CA with a content of CO more than 20% can be correctly identified by principal component analysis or partial least squares discriminant analysis, and the blended oils could be successfully classified by orthogonal partial least squares discriminant analysis, with an accuracy of 100% when the adulteration ratio was above 30%. These results indicate the practicability of LF-NMR in the rapid screening of food authenticity.

## 1. Introduction

Camellia oil (CA), obtained from camellia oleifera tree seeds, is one of the oldest edible wooden vegetable oils in China [1]. China has the largest concentration of camellia plants in the world, and camellia oleifera has been widely cultivated in southern regions of China such as Jiangxi, Zhejiang, Hunan and Fujian, accounting for about 90% of the global total [2]. The golden color, fragrant smell and pure taste, as well as its content of a variety of natural antioxidants, such as squalene, phytosterols, polyphenols, fat-soluble vitamins (vitamin A, vitamin B, vitamin E) and other functional substances[3], give CA attractive cosmetic and edible values. Moreover, its many health-promoting constituents, especially oleic acid and ω-6 linoleic acid, are deemed attractive materials for the production of functional foods or pharmacological supplements [4], which will give help in reducing cholesterol and preventing hypertension, heart disease, and other diseases. In addition, it can also be further processed as the base oil of advanced skin care oils [5]. Oleic acid, with a high fatty acid ratio, contributes to the major health benefits of CA for several human chronic diseases [6]. Therefore, CA is also called “Oriental olive oil” [7].

As a pure natural wooden vegetable oil promoted in China, and a healthcare edible vegetable oil first promoted by the Food and Agriculture Organization (FAO), CA is popular in China and some other Asian countries, and its price is usually 3–5 times higher than that of other ordinary edible oils [8]. Therefore, CA is often adulterated with other, cheaper oils for high profits by illegal traders [9], which directly deteriorates the inherent nutritional quality of CA and has a negative impact on health. Furthermore, the long-term mislabeling of CA derived from various botanical origins, geographical origins and extraction methods may lead to confusion in the local edible oil market, and endanger the development of international trade [10]. Therefore, it is extremely important to develop effective analytical methods for the quality evaluation and authentication identification of CA.

Besides the physical analysis methods, such as dielectric constant [11] and sensory evaluation [12], chromatography and mass spectrometry techniques [13,14] with high specificity and sensitivity [15] have also been popularized in the quality detection and safety assessment of edible oils. However, the further authentication detection of edible oil is often limited by time-consuming, destructive and extensive sample preparation. So as to overcome the above shortcomings, quite a few high-accuracy, fast and nondestructive spectral analysis methods, including near/mid-infrared spectroscopy (NIR/MIR), Fourier transform (FT)–Raman spectroscopy, nuclear magnetic resonance (NMR) spectroscopy, and fluorescence spectroscopy combined with chemometrics, have been selected as the preferred techniques for the quality assessment of CA [16,17]. In addition, a variety of feasible analytical methods, such as isotope ratio mass spectrometry (IRMS), ion mobility spectroscopy (IMS), differential scanning calorimetry (DSC), and electronic nose (e-nose) have also shown great potential in product certification [18].

As a non-destructive detection technique, NMR provides excellent repeatability and reproducibility, and can accurately quantify and provide structural information of the different compounds in a mixture [19,20,21]. Recently, NMR techniques, including high-field/low-field NMR (HF-/LF-NMR), have been used to detect CA components [22], measure the physical and chemical properties of cold-pressed and commercial refined CA [23], classify vegetable oils, and detect adulteration [24]. Moreover, the combination of NMR spectroscopy and multivariate statistical analysis offers an advantage in monitoring food quality and identifying food origins [7,25]. As a fast, simple and low-cost technique, LF-NMR has been applied to determine water distribution in food [26,27,28] and identify the edible oil species, its adulteration, and its origin [29,30,31]. However, most of these studies have focused on the detection of some specific components in CA or olive oil (OL), and there are few reports on the compositional differences between CA and OL, and especially the identification of CA adulteration. In this study, the nutritional compositions of CA and OL from different origins were investigated by HF-NMR technology, and their T_2_ relaxation profiles were assessed by LF-NMR. In addition, a pattern recognition model based on the relaxation time was established to quickly identify CA adulterated with corn oil (CO). The main aim of this study was to investigate the feasibility of using LF-NMR technology for the rapid identification of adulterated edible oils.

## 2. Results and Discussion

### 2.1. Nutritional Compositional Difference between CA and OL

**Figure 1:**
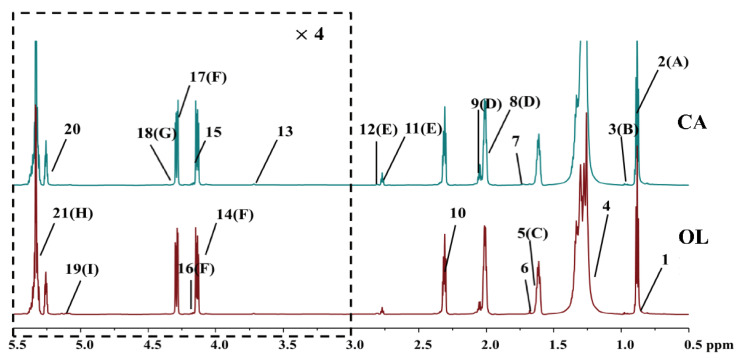
Representative 850 MHz ^1^H NMR spectra (δ0.5–5.5) of camellia oil (CA) and olive oil (OL). For clarity, the spectra in the region δ3.0–5.5 (in the dashed box) were magnified 4 times and scaled in a different chemical shift expansion compared with the region δ0.5–3.0. The detailed spectral information of the assigned and labelled signals is shown in Table S1.

Representative 850 MHz ^1^H NMR spectra of CA and OL are shown in Figure 1. A total of 21 components with high intensities were assigned according to the published reference [32] and have been labelled on NMR spectra, and the detailed spectra information are shown in Table S1 in the Supplementary Material. The NMR spectra have been divided into two regions in order to better and more clearly display all signals, as shown in Figure 1. The spectral characteristics of CA and OL were shown to be similar due to the predominant peaks of triglyceride in the whole spectrum. As shown in Figure 1 and Table S1, CA and OL are generally composed of saponifiable and unsaponifiable components. Saponifiable substances accounted for 98.5–99.5% of the vegetable oil, mainly including various fatty acid glycerides, and the unsaponifiable ones accounted for 0.5–1.5% of the vegetable oils, mainly including phytosterols, tocopherols, squalene, and pigments [33].

According to the superimposed NMR spectra of different brands of CA and OL, the relative content of (CH_2_)_n_ in all fatty acids of “DL” CA at δ1.30 was apparently different from other brands, and the relative content of squalene at δ1.82 in CA was obviously higher than that in OL (Figure S1). Squalene, also referred to as shark terpene and cod liver oil, is considered to be an essential and highly functional biologically active substances [34], and it can perform favorable functions in purifying blood, strengthening the liver, activating the body’s functional cells, disinfecting and sterilizing, and beautifying the skin [35,36] as an unsaturated hydrocarbon. The higher relative content of squalene in CA may imply the higher nutritional value of CA.

Differences in nutritional components between CA and OL were further determined by comparing the integral area of the corresponding signals of any component with that of the internal standard (TMS), with spin–lattice relaxation time (T_1_) correction. Accordingly, the relative concentrations of all kinds of components in the CA and OL are listed in Table 1. The results show that the principal nutritional components in CA, such as linolenic acid, oleic acid, saturated fatty acid and hydroxyl value, are similar to those of OL, which is consistent with our previous study [32]. However, the linolenic acid (2.16 ± 0.53%) and iodine value (82.04 ± 1.02) in CA were slightly higher than those in OL, while the acid value showed the opposite (CA: 0.72 ± 0.59%, OL: 1.18 ± 0.35%). In our previous study [32], the contents of oleic acid in CA and OL were much higher than in other edible oils, such as CO, soybean oil (SO), and sunflower seed oil (SS), which indicates that CA and OL have more nutritional value than these others. Meanwhile, in terms of minor components, the contents of squalene and other active substances in CA were higher than those in OL, indicating the higher nutritional value of CA than OL.

As a polyunsaturated Ω-9 fatty acid, oleic acid reduces the synthesis of low-density lipoprotein (LDL) in the human body, promotes the increase in high-density lipoprotein (HDL), and regulates blood lipids and cholesterol in the body, which effectively reduces the risk of common chronic diseases (CVDs) such as cancer, diabetes and cardiovascular disease. Oleic acid can also be used to treat patients with severe hyperirritability and inflammation and improve cortical dysfunction, which also has a vital role in monounsaturated fatty acids [32]. As a polyunsaturated Ω-3 fatty acid, linolenic acid is an essential fatty acid for humans. Other than the above-mentioned oleic acid-like effects, such as lowering blood lipids and preventing infarctions and other diseases, it also has the healthcare function of protecting the eyes and improving eyesight [37]. The higher iodine value indicated that the oil sample contained more carbon–carbon double bonds (C=C) in the unsaturated fatty acids, that is, the higher the content of unsaturated fatty acids [38,39], the more beneficial to human health.

As shown in Table 1, the content of linolenic acid was significantly different (*p* < 0.05) between CA and OL, while no significant difference was observed in other components, such as linoleic acid, oleic acid and saturated fatty acid. In the fatty acid evaluation indexes, acid value and iodine value were significantly different between CA and OL. The content of unsaturated fatty acids in CA was significantly higher than that in OL, while the content of free fatty acids was lower than that in OL. Therefore, it could be considered that the two kinds of high-quality edible oils demonstrate similar, but partially different, main nutritional components. As CA and OL came from different provinces in China and different countries in Europe, it was found that geographical origins would affect the content proportions of nutrients in the same vegetable oil, which is consistent with the results in the previous study [10]. Furthermore, the contents of squalene, oleic acid, linolenic acid and other unsaturated fatty acids in CA were significantly higher than that in OL.

### 2.2. T_2_ Distribution of CA and OL

**Figure 2:**
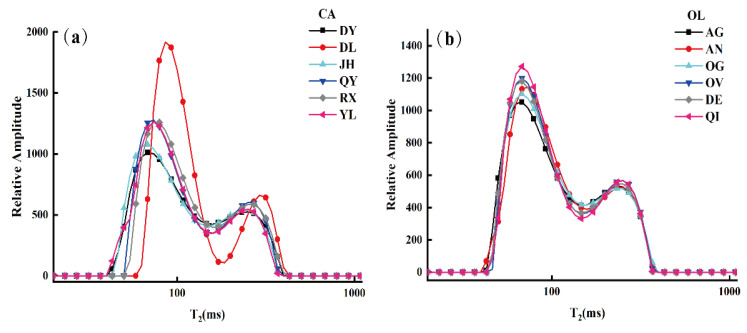
T_2_ distribution of different brands of camellia oil (CA) (**a**) and olive oil (OL) (**b**).

For exploring the detailed regularity between oil samples, the transverse relaxation time (T_2_) distributions of CA and OL were measured by LF-NMR. Figure 2 displays the distribution of different brands of CA and OL in the transverse relaxation time (T_2_), where the T_2_ distribution curves show the average spectrum of oil samples. As shown in Figure 2, the relaxation spectra showed similar spectral characteristics with two strong peaks, T_21_ and T_22_ (left peak and right peak, respectively), indicating that there were two different hydrogen proton components in the fatty acid chain of the sample with different transverse relaxation times. This may be attributed to the similarity of triacylglycerol (TGs) components and the similar chemical environment of the hydrogen protons in these oil samples [24,40,41]. Furthermore, there was some variation among the peak amplitudes and positions of the different brands of oil, which might be attributed to the esterification reaction of fatty acids, constituted by a 12–18-carbon chain in edible oil (including saturated and unsaturated fatty esters). In the multi-fatty ester system, the increase in carbon chain length brought about a decrease in T_2_ and ($\bar{T_{2w}}$), but the opposite result appeared for the variety of unsaturation [38]. It should be noted that the relaxation spectra of CA from “DL” were quite different from those of other samples in relative amplitude (i.e., the amplitude of T_21_ and T_22_). The reason for this was perhaps that the content of fatty acids in “DL” CA was explicitly different from the proportion of fatty acids in other brands of CA (as shown in Table 1).

The transverse relaxation parameters of the samples (i.e., $\bar{T_{2w}}$, T_21_ and T_22_) and the integral area percentages of different components (i.e., S_21_, S_22_) are summarized in Table 2 in order to visualize the T_2_ distribution of different brands of CA and OL. The results show that there were significant differences in terms of parameters between “DL” CA and the other brand of CA, and there were no significant differences in the T_2_ distribution parameters of the same kind of edible oil.

Student’s *t*-test was carried out to compare the differences in T_2_ distribution parameters between CA and OL for further analysis. As shown in Table 2, the *p* values of T_21_ and $\bar{T_{2w}}$ were 0.023 and 0.049, respectively. The significant difference in T_21_ between the CA and OL suggests a significant difference in more stable hydrogen protons in CA and OL. This might be due to some differences in fat acids, such as linolenic acid. However, no statistical differences were observed in other parameters, indicating a similar profile in these parameters between CA and OL. It is necessary to point out that the weighted average horizontal time $\bar{T_{2w}}$ was calculated by Equation (1), and the value of $\bar{T_{2w}}$ was proportional to T_21_ and T_22_, but inversely proportional to the integral area of S_21_ and S_22_, which can better reflect the T_2_ relaxation characteristics of CA and OL. The *p* value of $\bar{T_{2w}}$ between CA and OL approached 0.05 (*p* = 0.049), which proved that the $\bar{T_{2w}}$ values of CA and OL were different, but it is not obvious. This may be attributed to the fact that the types and contents of fatty acids in CA and OL were similar, as shown in Table 1 (obtained by HF-NMR).

**Figure 3:**
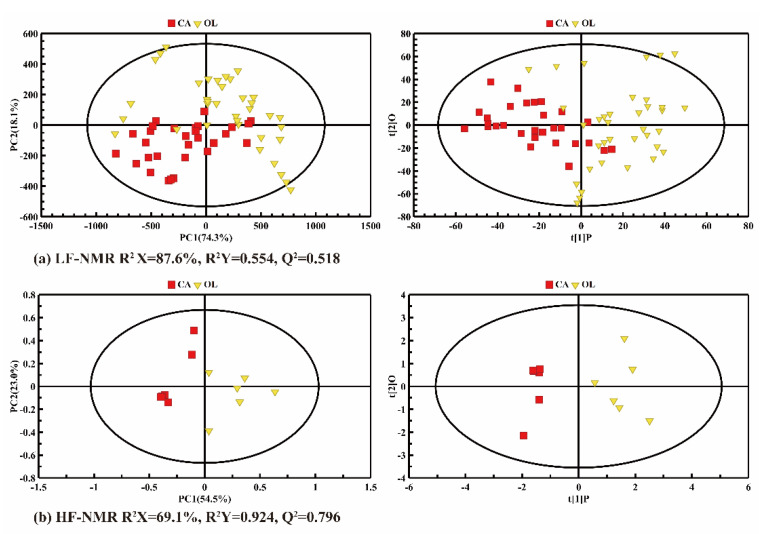
The PCA (left panel) and PLS-DA (right panel) score plots of the camellia oil (CA) and olive oil (OL) based on LF-NMR data (**a**) and HF-NMR data (**b**).

A global PCA and PLS-DA were conducted on the LF-NMR (Figure 3a) and HF-NMR data (Figure 3b) of CA and OL to evaluate their potential classification. In the PCA score plots in Figure 3 (left panel), the distribution of variability can be mainly explained by the two principal components, contributing about 92.4% and 77.5%, respectively. As analyzed by LF-NMR data (Figure 3a), some overlaps appeared in PCA and PLS-DA, indicating that the relaxation parameters of CA and OL were similar, and detailed differences could not be detected by LF-NMR. As shown in Figure 3b, the CA and OL samples could be clearly distinguished, indicating that the difference in composition between CA and OL could be detected by HF-NMR.

### 2.3. Identification of CA Adulteration by LF-NMR

#### 2.3.1. T_2_ Distribution of the Adulterated CA

**Figure 4:**
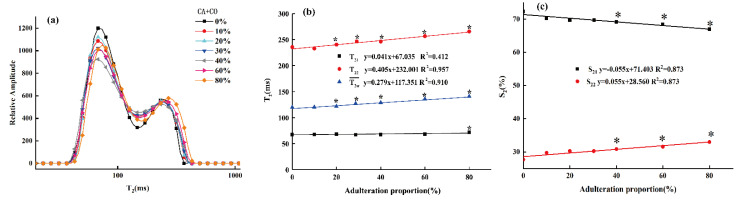
T_2_ distribution of camellia oil (CA) adulterated with various proportions of corn oil (CO) (**a**); LF-NMR parameter changes of camellia oil (CA) samples adulterated with different adulteration ratios of corn oil (CO). The transverse parameters of the single component’s (weighted average) relaxation time $\bar{T_{2w}}$, the multi-component transverse relaxation times T_21_ and T_22_ (**b**), and the peak area proportions S_21_ and S_22_ (**c**). * represents a significant difference (*p* < 0.05) between the pure camellia oil (CA) and the samples at specific adulteration ratios.

To explore the detailed regularity of the adulterated oil samples, their T_2_ distributions were investigated by LF-NMR. As shown in Figure 4a, the T_21_ and T_22_ peaks’ amplitude in the average relaxation spectra varied with the different levels of CO adulteration, though they showed similar spectral profiles. From the results of the HF-NMR analyses, the content of unsaturated fatty acids in CA was higher than that in OL, while our previous research showed that the content of unsaturated fatty acids in OL was significantly higher than that in SO and CO [32]. Therefore, it can be inferred that the unsaturated fatty acid content of CA was higher than that of CO. This difference in fatty acids content is also the reason why the T_2_ relaxation spectra of adulterated oil with various concentrations of CO are different from those of pure CA [42]. Furthermore, as the proportion of adulterated CO increased, the T_2_ distribution curve tended to shift to the right. This was because, as the proportion of oleic acid increased, the nuclear magnetic response of the mixed system was gradually dominated by oleic acid containing double bonds, the intermolecular hydrogen bonds and van der Waals forces and other forces were relatively weakened, and the molecular structure was relatively loosened. The non-uniformity of the response of hydrogen protons in the magnetic field increased, and the relaxation response time increased as well [38].

To deeply explore the influence of different proportions of CO on the T_2_ distribution of CA, the transverse relaxation parameters ($\bar{T_{2w}}$, T_21_ and T_22_) of the adulterated oil samples and the integral area ratios of different components (S_21_, S_22_) were analyzed, as shown in Figure 4b,c.

In Figure 4b, the horizontal and vertical axes represent the adulteration proportions and the transverse relaxation time ($\bar{T_{2w}}$, T_21_ and T_22_), respectively. As the proportion of adulterated CO increased, the weighted average transverse relaxation time (i.e., single-component relaxation time, $\bar{T_{2w}}$) of adulterated CA increased linearly (the linear correlation value is 0.910). Moreover, the multi-component transverse relaxation time (T_21_, T_22_) also increased linearly with the adulteration ratio, although the T_22_ increased more obviously, while the T_21_ changed relatively gently. This might be due to the similar composition of TGs in the oil samples, meaning the transverse relaxation times (T_21_, T_22_) were not significantly different from those of pure CA samples [24,43]. As shown in Figure 4c, the increase in adulteration ratio led to significant differences in the peak area ratios (S_21_, S_22_) between adulterated CA and pure CA samples. In each adulterated oil sample, the S_21_, which represents the number of relatively stable hydrogen protons, was higher than that of S_22_, which represents the number of unstable hydrogen protons. In addition, the S_21_ of the adulterated CA samples decreased linearly with the increase in the adulteration ratio, but the S_22_ showed the opposite trend, indicating that the unstable hydrogen protons in the samples decreased with the increase in the adulteration ratio. The results show that the transverse relaxation time distribution, the T_2_ parameter and the peak integral ratio of different components of the adulterated CA were similar to those results in our previous studies on OL adulterated with SO and CO [44]. This verifies that CA, known as “Oriental olive oil”, has a similar fatty acid composition to OL.

#### 2.3.2. Identification of Adulterated CA with Pattern Recognition Analysis

**Figure 5:**
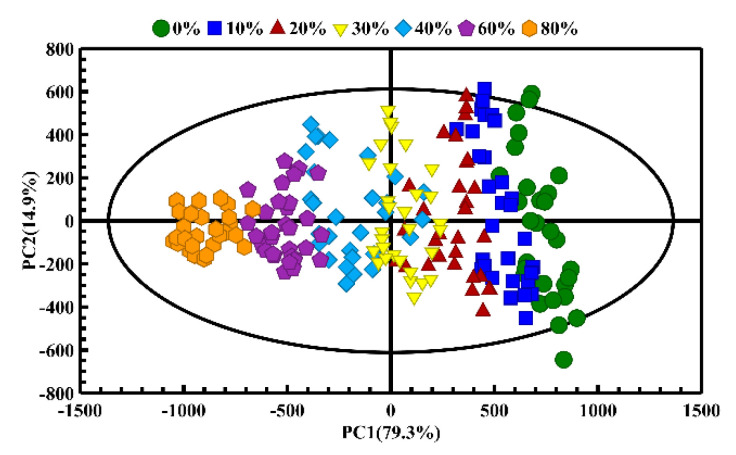
The PCA score plots of the camellia oil (CA) adulterated with different adulteration ratios of corn oil (CO).

PCA was firstly used to monitor the distinction between pure CA and CA adulterated with different proportions of CO to understand the adulteration profiles. As shown in Figure 5, when CA was adulterated with different proportions of CO, the distribution of variability can be mainly explained by the first two principal components (containing 94.2% of the variance in the original data). The PCA score plots show the obvious separation between different adulterated oil samples via the classification trajectory. The pure CA of different brands can be seen on the right side of the first principal component (PC1), while the adulterated oil samples deviated from the pure CA with the increase in the adulteration proportion, and the adulterated oils regularly moved along the negative direction of the PC1 axis (i.e., from the right and left). Further analysis found that the oil samples in lower adulteration ratios (10%) and pure CA overlapped with each other, and the groups from the adjacent adulteration ratio had a certain overlap, which might be due to the similar fatty acid compositions and component contents of the oil samples in the lower adulteration ratio [32].

It is necessary to point out that the adulterated oil samples tended to be more clustered with the increase in the proportion of CO adulteration. This behavior may be caused by the slight differences in fatty acid content and T_2_ distribution of different brands of pure CA, which has been proven by the LF- and HF-NMR studies. The CA samples of different brands were observed to separate from each other, but the oil samples showed an intra-group cluster and inter-group differentiation in PCA score plots in general. With the increase in adulteration ratio of CO, the fatty acid compositions of CO held superior influence over that CA. Therefore, the oil samples in different adulterated ratios show an obvious cluster in the PCA score plots as the CO adulteration is increased. When the CO adulteration rate exceeded 20%, the pure CA and adulterated CA samples could be clearly distinguished, as shown in the PCA score plot (Figure 5).

**Figure 6:**
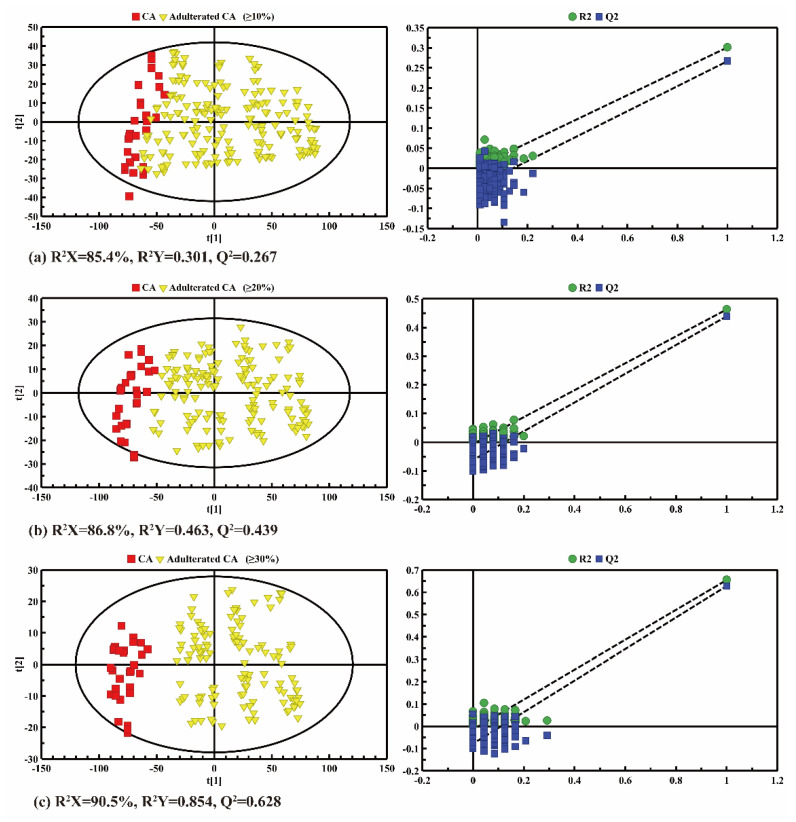
PLS-DA score plots (left panel) and cross validation plots (right panel) by permutation test (*n* = 200) between the pure camellia oil (CA) and the CA adulterated with different adulteration ratios ranging from (**a**) 10% to 100%; (**b**) 20% to 100% and (**c**) 30% to 100%.

Pairwise comparisons between pure CA and adulterated CA were conducted by PLS-DA in order to further identify the adulteration. The PLS-DA results of pure CA and adulterated CA with 10%, 20% and over 30% CO are shown in Figure 6. As shown in the score plots (left panel in Figure 6), the distinction between pure CA and adulterated CA became more and more obvious with the increase in the adulterated proportion of CO. When the adulteration ratio of CO was 30−100%, the pure and adulterated CA were well separated, and the predicted values of Q^2^ were reasonable (both greater than 0.400), which indicates the strong predictability of the model and the reliable previous analysis of the fatty acids’ compositions. Furthermore, the increase in the adulteration rate would also lead to an increase in the R^2^Y and Q^2^ values of the model, for example from CA-adulterated CA (≥10%) (R^2^Y = 0.301, Q^2^ = 0.267) and CA-adulterated CA (≥20%) (R^2^Y = 0.463, Q^2^ = 0.439) to CA-adulterated CA (≥30%) (R^2^Y = 0.854, Q^2^ = 0.628). The cross-validation permutation test also showed a similar trend (right panel in Figure 6), in which the steeper the regression line was, the better the LF-NMR data fitted the model for R^2^Y, and the more significant the composition differences were between the fatty acids. The big difference between R^2^ and Q^2^ might indicate that the model was over-fitted, and the difference between oil samples with different adulteration rates is not clear. Obviously, the PLS-DA results show that pure CA and adulterated CA can be clearly distinguished when the adulteration rate is beyond 20%.

#### 2.3.3. Prediction of Adulterated CA by Discriminant Analysis 

**Figure 7:**
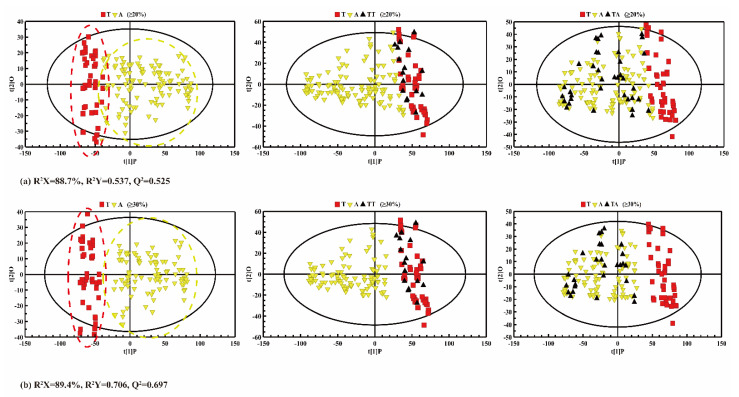
OPLS-DA score plots for discrimination analysis with adulteration ratios ranging from (**a**) 20% to 100%; (**b**) 30% to 100%. Left panel: training data set between the true camellia (CA) and the adulterated CA. Middle panel: the prediction of the true CA. Right panel: the prediction of adulterated samples (T: true CA; A: adulterated CA; TT: test set of true CA; TA: test set of adulterated CA).

From the PCA (Figure 5) and PLS-DA (Figure 6) results, it is difficult to accurately distinguish the adulterated oil samples from pure CA when the adulteration rate is 10%. As a result, the oil samples with a 10% adulteration ratio of CO were artificially classified as true CA in the following predictability analysis. Figure 7 shows an OPLS-DA score plot (left panel) for the adulteration predictability analysis, showing that both true CA and adulterated CA samples cluster and distribute within 95% confidence intervals. The parameters of Q^2^ and R^2^ also indicate that there were significant differences between the true CA group and the adulterated CA group (i.e., R^2^Y = 0.537, Q^2^ = 0.525 in Figure 7a and R^2^Y = 0.706, Q^2^ = 0.697 in Figure 7b).

However, the adulterated CA samples overlapped slightly with the pure CA, and cannot be clearly discriminated (left panel in Figure 7a) when the adulteration was more than 20%. After eliminating the 20% adulterated CA samples, the CA samples with a 30~100% adulteration rate can be clearly distinguished from the pure CA samples (left panel of Figure 7b).

To calculate the prediction rate of the model, the data of the testing set were imported into the OPLS-DA model, and the prediction results of the established model are shown in Figure 7b,c. In Figure 7a, the middle panel is the prediction of pure CA samples, and the right panel is the prediction of adulterated CA samples. All the prediction results are summarized in Table 3. When the adulteration ratio was 20–100%, there were still a few errors in the discrimination of the sample in the prediction set (Figure 7a). However, as the adulteration ratio was 30–100%, the pure CA and the adulterated CA could be clearly distinguished in both the training set and the prediction set. As shown in Table 3, even though the adulteration ratio was 20%, the correct classification rates of the pure CA group could reach 84.1% (calibration) and 68.8% (validation), the correct classification rates of the adulterated CA group were 90.3% (calibration) and 85.7% (validation), and the total classification rates for the calibration and validation samples were 88.6% and 79.5%, respectively. This might be due to the fact that the content of fatty acids in the 20% adulterated CA was slightly similar to that of the pure CA, so OPLS-DA analysis could not distinguish them clearly. Nevertheless, the accuracy of all predictions can reach 100% when the adulteration ratio is over 30%, which suggests the feasibility of discriminating pure CA from the adulterated ones. The same proportion of CA adulteration (30%) is more accurate here than in the latest research [45], the classification rate of which is 92.31%. These results coincide with a previous study on adulterated OL and adulterated CA using LF-NMR [44].

All in all, our results show that the ^1^H LF-NMR technique, combined with the discriminant analysis method, could be used to determine the authenticity of CA rapidly and simply. For the multi-blended oil mixture classification, as the adulteration ratio was greater than 30%, the correct classification rate of CA adulterated with CO was 100%.

## 3. Materials and Methods

### 3.1. Experimental Oil Sample

Different brands of CA (coded DY, JH, QY, RX, YL, and DL) and OL (coded AN, AG, OG, OV, DE and QI) from different geographical origins were provided by the Technology Center of Xiamen Customs. CO samples were purchased from the local supermarkets. The sample information is shown in Table S2 in the Supplementary Material. All the CA, OL, and CO samples were stored in a dark environment with air conditioning at 20 °C before the experiment.

#### 3.1.1. Sample Preparation for HF-NMR

Before HF-NMR analysis, 150 μL of the CA or OL samples was dissolved in 600 μL of CDCl_3_ (containing 0.03% sodium 3-(trimethylsilyl) propionate-2,2,3,3-d4 (TMS)) and oscillated and vortexed for 60 s and held at 25 °C for 5 min. Then, 600 μL of the oil samples were transferred to a 5 mm NMR tube, and kept at 4 °C until HF-NMR analysis.

#### 3.1.2. Sample Preparation for LF-NMR

In order to prepare the binary blend samples for LF-NMR measurement, CO was added into 6 different brands of CA at volume ratios of 0%, 10%, 20%, 30%, 40%, 60% and 80%. After vortexing, 1.0 mL of each experimental sample was extracted and transferred into a 10 mm NMR tube. Each sample at the same adulteration ratio was prepared in triplicate with double repeated detection to reduce the measurement error. Thus, a total of 126 samples were obtained for LF-NMR.

### 3.2. NMR Detection

#### 3.2.1. HF-NMR Detection 

All ^1^H NMR spectra of CA and OL were acquired at 298 K with an 850 M Hz Bruker Advance III HD NMR spectrometer (Bruker Corporation, Karlsruhe, Germany) equipped with a 5 mm triple resonance TCI reverse ultra-low temperature probe operating at 850.29 MHz. The specific detection parameters were as follows: The zg30 pulse sequence was used for data acquisition of experimental oil samples. The 90° pulse was adjusted to 7.5 μs at 298 K. The numbers of sampling accumulations (NS) and relaxation delays (D1) were 32 and 5 s, and the spectral width (SW) and gain (RGA) were set to 12 kHz and 2, respectively.

#### 3.2.2. LF-NMR Detection 

The relaxation time curves of all the adulterated CA samples were measured on an EDUMR20-015V-I NMR spectrometer (Suzhou Niumai Analytical Instrument Co., Ltd., Suzhou, China), equipped with an RF coil probe with a diameter of 18 mm and a magnetic field strength of 0.53 T. The pulse sequence and its parameters were as follows: Carr-Purcell-Meiboom-Gill (CPMG) pulse sequence was used to measure the transverse relaxation time (T_2_), the 90° pulse was 13 μs, the experimental temperature was 35 °C, the number of scans was 4, and the number of echo data points was 8000. The sampling frequency (SW) and re-sampling wait time (TW) were 250 kHz and 5000 ms, respectively, and the interval between 90° and 180° pulses was 200 μs.

### 3.3. Preprocessing of NMR Spectra

#### 3.3.1. HF-NMR Spectral Preprocessing 

The ^1^H NMR spectra preprocessing was performed on the software of MestReNova (V9.0.1, Mestrelab Research S.L., Santiago de Compostela, Spain). All the free induction decays (FIDs) were zero-filled to 64 k data points and multiplied by an exponential function with a line-broadening factor of 0.5 Hz before the fast Fourier transformation. Then, the spectra were also manually phased and baseline-corrected to overcome the spectra distortion. The internal standard TMS resonance at 0.0 ppm served as a standard reference for the chemical shifts in oil samples. The spectral regions after δ5.5 were removed, and the spectral regions between δ0.50 and δ5.50 were retained.

The spectral peaks of CA and OL in the HF-NMR spectra were assigned according to the published literature [32,46,47]. Each spectrum was then binned into 2500 buckets across the range of δ5.5–0.5. The bucketed data of each spectrum were normalized by the method of probabilistic quotient normalization (PQN) to reduce the influence of the content difference between the samples [48]. Finally, the preprocessed NMR data were exported for further nutrient composition calculation and pattern recognition analysis.

#### 3.3.2. LF-NMR Spectral Preprocessing 

All LF-NMR transverse relaxation data were inverted into T_2_ distribution on the window analysis platform using the multi-exponential fitting analysis (T-invfit) program, which was also used to calculate the relaxation time of each component in the oil and the amplitude and area of the relaxation time peak. Then, the peak value within the relaxation time range (0.0–1600 ms) of the T_2_ curve of each oil sample was taken as the benchmark for normalization and subsequent analysis.

Hence, the weighted average of each peak’s T_2_ can be obtained by (1):

(1) $$\bar{T_{2w}}=\sum_{i=1}^{n}\frac{S_{i}}{\sum_{i=1}^{n}S_{i}}T_{2i}$$

where *T*_2*i*_ and $\bar{T_{2w}}$ correspond to the *i*th transverse relaxation time and the geometrically weighted average of the transverse relaxation time, respectively; *S_i_* was the integral area value of the *i*th relaxation time.

### 3.4. Quantitative Calculation of Nutritional Components of Oil Samples

After the data preprocessing of the ^1^H-NMR spectra of CA and OL, the content percentages of different nutrients in CA and OL could be obtained according to Equations (2)–(8). Meanwhile, three fatty acids’ evaluation indices, acid value, iodine value and hydroxyl value in edible oil were introduced according to the equations, as follows:

[Linolenic acid] = B/(A + B)(2)

[Linoleic acid] = (3E − 4B)/[2(A + B)](3)

[Oleic acid] = 3D/[4(A + B)] − [Linolenic acid] − [Linoleic acid](4)

[Saturated fatty acid] = A/(A + B) − [Linoleic acid] − [Oleic acid](5)

[Iodine value] = (H/2 − G/4)/[(A + B)/3] × 86(6)

[Acid value] = (C/2 − 3F/4 − G/2)/(C/2) × 100% × [Average molecular weight]/56(7)

[Hydroxyl value] = I/[4(A + B)](8)

where A, B, C, D, E, F, G, H and I are the ascribed integral values of the corresponding peaks in Table S1, respectively. The acid value represents the content of KOH consumed to neutralize the free acid in 1 g of fat, which reflects the amount of free fatty acid in the edible oil.

### 3.5. Multivariate Statistical Analysis

The NMR data were imported into SIMCA (version 14.1, Umetrics AB, Umea, Sweden) for multivariate statistical analysis. Firstly, the normalized data were scaled by mean centering (Ctr), and principal component analysis (PCA) was performed to summarize the data distribution and identify potential outliers. Then, partial least squares discriminant analysis (PLS-DA) and orthogonal partial least squares discriminant analysis (OPLS-DA) under a PAR scaling pattern were conducted to investigate component differences between the pure CA group and the OL group for a better understanding of the specific differences between the pure CA group and the adulterated CA group. The model was verified by 7-fold cross validation, permutation tests (permutation number = 200) and cross validation-analysis of variance (CV-ANOVA). A calculated *p* value of less than 0.05 was considered to be statistically significant.

### 3.6. Discriminant Analysis

In total, 81 adulterated CA samples (i.e., 3/4 of the 108 samples) were randomly selected as the training set, and the remaining 1/4 samples were set as the prediction set. The prediction set of the OPLS-DA model was established to detect and discriminate accuracy, and the threshold value was set at 0.5. The value of pure CA (i.e., 0% adulterated ratio) was set as 1, and the value of pure CO (i.e., 100% adulterated ratio) was set as 0. When the predicted value was greater than 0.5, the sample was determined to be pure CA, otherwise it was determined to be adulterated CA. Moreover, the Origin software (version 9.1, OriginLab, Northampton, MA, USA) was used for data fitting and the linear regression analysis of T_2_ distribution.

## 4. Conclusions

In this study, HF and LF-NMR techniques combined with multivariate statistical analysis were used to explore the nutritional differences between CA and OL, and the relaxation time distribution of pure CA and adulterated CA. The HF-NMR spectra showed similar but partially different fatty acid profiles between CA and OL. The contents of oleic acid and linolenic acid, as well as the iodine value, of CA are all higher than those of OL. Besides this, squalene, a high-quality bioactive and healthy substance, is also present at a higher content in CA than in OL, indicating that the nutrients in CA are higher than those in OL. Furthermore, owing to the similarity of fatty acid composition and magnetic sensitivity, the T_2_ relaxation parameters of LF-NMR are similar between CA and OL. The PCA models (based on CA+CO samples) successfully accomplished the intra-group gathering and the inter-group distinction. Moreover, pure CA and binary adulteration CA with an appropriate adulteration ratio (no less than 30%) could be clearly discriminated by OPLS-DA models. Our results suggest that LF-NMR technology combined with pattern recognition analysis can be extensively used as a fast, efficient and convenient screening technique for the adulteration identification of edible oil.

## Figures and Tables

**Table 1:** Relative contents of main components and evaluation indexes in camellia oil and olive oil, as determined by high field NMR.

Types of Oil Sample		Linolenic Acid (%)	Linoleic Acid (%)	Oleic Acid (%)	Saturated Fatty Acid (%)	Acid Value (%)	Squalene (%)
Camellia oil	DY	2.54	6.51	75.31	15.64	0.36	3.45
	JH	1.03	5.08	81.42	12.47	0.45	2.00
	QY	2.37	5.45	76.69	15.50	0.19	3.96
	RX	2.60	6.31	75.27	15.82	0.55	1.88
	YL	2.34	4.31	77.15	16.21	0.76	1.69
	DL	2.09	5.08	77.02	15.81	1.98	1.23
	Mean ± SD ^a^	2.16 ± 0.53	5.46 ± 0.76	77.14 ± 2.06	15.24 ± 1.26	0.72 ± 0.59	2.36 ± 0.98
Olive oil	AN	2.03	5.53	77.17	15.26	1.75	0.78
	AG	1.56	8.12	72.99	17.33	0.85	0.27
	OG	1.07	8.17	71.12	19.65	0.98	0.53
	OV	2.17	2.63	77.38	17.81	1.50	0.51
	DE	2.10	5.01	74.18	18.71	1.21	0.49
	QI	1.42	5.12	77.55	15.91	0.77	0.36
	Mean ± SD	1.73 ± 0.41	5.76 ± 1.92	75.07± 2.47	17.44 ± 1.51	1.18 ± 0.35	0.49 ± 0.16
*p* values		0.014	0.827	0.364	0.057	0.005	0.002

^a^ SD: standard deviation.

**Table 2:** T_2_ distribution parameters of camellia oil and olive oil.

Types of Oil Sample		T_21_ (ms)	T_22_ (ms)	$\bar{T_{2w}}$ (ms)	S_21_ (%)	S_22_ (%)
Camellia oil	DY	69.38 ± 2.35	241.63 ± 4.27	119.88 ± 3.25	70.68 ± 0.46	29.32 ± 0.46
	DL	87.35 ± 2.96	304.21 ± 11.67	137.00 ± 7.37	77.17 ± 1.43	22.83 ± 1.46
	JH	68.02 ± 2.65	241.63 ± 3.27	121.18 ± 2.43	69.43 ± 0.92	30.57 ± 0.92
	QY	73.45 ± 2.53	250.90 ± 4.22	124.75 ± 0.73	70.98 ± 0.41	29.02 ± 0.41
	RX	76.38 ± 2.93	250.91 ± 3.54	129.54 ± 3.01	71.19 ± 0.12	28.81 ± 0.12
	YL	69.99 ± 4.47	243.05 ± 5.87	118.51 ± 5.54	71.69 ± 2.60	28.31 ± 2.60
	Mean ± SD ^a^	70.94 ± 5.98	246.12 ± 7.86	122.81 ± 4.91	70.90 ± 1.63	29.10 ± 1.63
Olive oil	AN	66.35 ± 1.37	238.54 ± 3.74	117.48 ± 2.35	70.32 ± 1.04	29.68 ± 1.04
	AG	70.74 ± 2.71	234.07 ± 2.32	114.53 ± 9.40	73.18 ± 0.23	26.82 ± 0.23
	OG	68.02 ± 2.33	232.36 ± 3.46	114.80 ± 2.83	71.53 ± 1.72	28.47 ± 1.72
	OV	68.02 ± 1.25	250.90 ± 2.54	122.59 ± 1.26	70.16 ± 0.69	29.84 ± 0.69
	DE	66.35 ± 2.37	244.72 ± 3.74	118.26 ± 3.02	70.90 ± 0.25	29.10 ± 0.25
	QI	68.02 ± 1.28	241.63 ± 4.27	118.01 ± 1.47	71.17 ± 0.75	28.83 ± 0.75
	Mean ± SD	67.67 ± 2.46	241.18 ± 4.31	117.61 ± 3.95	71.09 ± 1.28	28.91 ± 1.28
*p* values		0.023	0.359	0.049	0.694	0.694

^a^ SD: standard deviation.

**Table 3:** Discrimination accuracy of the OPLS-DA.

Adulteration Ratio	OPLS-DA Model	True Camellia Oil	Adulterated Camellia Oil	Total Accuracy
20–100%	Training set (*n* = 158)	84.1% (37/44)	90.3% (103/114)	88.6% (140/158)
	Prediction set (*n* = 44)	68.8% (11/16)	85.7% (24/28)	79.5% (35/44)
30–100%	Training set (*n* = 136)	100% (44/44)	100% (92/92)	100% (136/136)
	Prediction set (*n* = 36)	100% (16/16)	100% (20/20)	100% (36/36)
40–100%	Training set (*n* = 113)	100% (44/44)	100% (69/69)	100% (113/113)
	Prediction set (*n* = 29)	100% (16/16)	100% (13/13)	100% (29/29)

## Data Availability

Data are contained within the article and the Supplementary Materials.

## Supplementary Materials

The following are available online, Figure S1: The superimposed NMR spectra in the characteristic spectral regions of camellia oil (CA) and olive oil (OL). Table S1: Assignment of spectral peaks of camellia oil and olive oil in NMR. Table S2: Basic information of the experimental oil samples.

## Sample Availability

Samples of the NMR experiments are available from the authors.
