# Supplementary material for: Composition Profiling and Authenticity Assessment of Camellia Oil Using High Field and Low Field 1H NMR
Source: Molecules. 2021 Aug 5;26(16):4738. doi: 10.3390/molecules26164738 (PMC8400449; doi:10.3390/molecules26164738)
Supplement: Supplementary file 1 [file molecules-26-04738-s001.zip › molecules-1289427-supplementary.pdf]

Supplementary Material

Composition Profiling and Authenticity Assessment of Camellia Oil Using High Field and Low Field ^{1}H NMR

Meijun Xing [1+], Shenghao Wang [1+], Jianzhong Lin [2], Feng Xia [1], Jianghua Feng [1], and Guiping Shen [1, *]

[1] Department of Electronic Science, Fujian Provincial Key Laboratory of Plasma and Magnetic Resonance, Xiamen University, Xiamen 361005, China;

[2] Inspection and quarantine Technology Center of Xiamen, Xiamen 361012, China;

[+] These authors contributed equally to this study.

* Correspondence:

[1] 422 Siming South Road, Xiamen University, Xiamen, Fujian Province 361005, China.

Tel.: +86-592-2180728, Fax: +86-592-2181812.

Email address: gpshen@xmu.edu.cn (G. Shen)

Submitted to: ***Molecules***

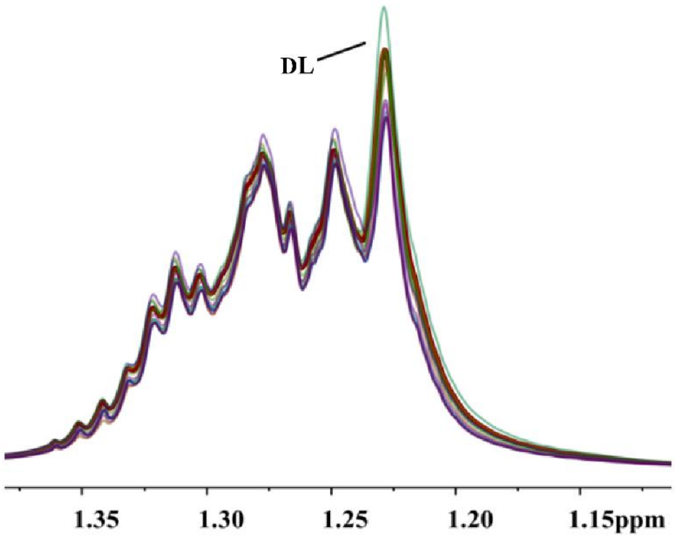

(a)

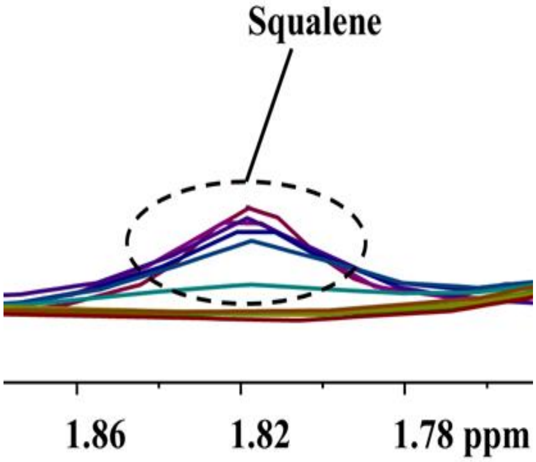

(b)

Figure S1 The superimposed NMR spectra in the characteristic spectral regions of camellia oil (CA) and olive oil (OL).

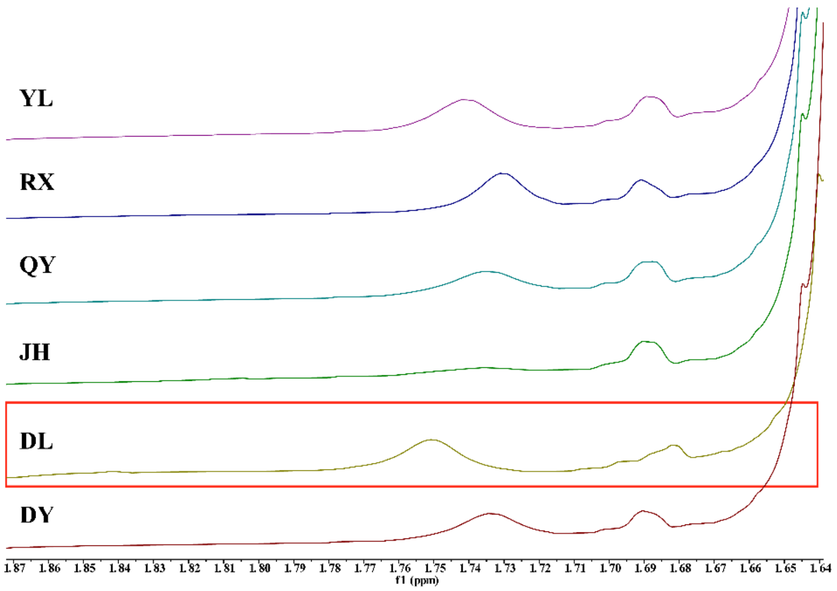

Figure S2 The partial spectrum of different brands of CA

Table S1 Assignment and spectral information of NMR spectral of camellia oil and olive oil

Peak No	Chemical shift ppm (multiplicity)	Proton	Assignment
1	0.81 (m)	H-24,30,29	β-sitosterol
2(A)	0.88 (br)	$CH_2CH_2CH_2$-C$\underline{H_3}$	All fatty acids (Except linolenic)
3(B)	0.97 (t)	CH = CH-CH_2-C$\underline{H_3}$	Linolenic
4	1.30 (br)	$(CH_2)_n$	All fatty acids
5(C)	1.60 (br)	CH_2-CH_2-COO-	All fatty acids
6	1.68 (s)	OH	Terpenes
7	1.82 (s)	–CH_3	Squalene
8(D)	2.00 (q)	C$\underline{H_2}$-CH = CH	Oleic
9(D)	2.05 (q)	C$\underline{H_2}$-CH = CH	UFA(Except oleic)
10	2.32 (m)	C$\underline{H_2}$-COO-	All fatty acids
11(E)	2.77 (t)	CH = CH-C$\underline{H_2}$-CH = CH	UFA(Except linolenic)
12(E)	2.81 (t)	CH = CH-C$\underline{H_2}$-CH = CH	Linolenic
13	3.73 (d)	–C$\underline{H_2}$OH	sn-1,2-Diglycerides
14(F)	4.14 (dd)	C$\underline{H_2}$-OCOR sn-1,3	Triacylglycerols
15	4.18 (dd)	C$\underline{H_2}$-OCOR, CH_2OH sn-1,3	sn-1,2-diacylglycerols
16(F)	4.23 (dd)	C$\underline{H_2}$-OCOR sn-1,3	sn-1,3-diacylglycerols
17(F)	4.31 (dd)	C$\underline{H_2}$-OCOR sn-1,3	Triacylglycerols
18(G)	4.37 (dd)	C$\underline{H_2}$-OCOR sn-1,3	sn-1,3-diacylglycerols
19(I)	5.12 (m)	C$\underline{H}$OH	sn-1,2-diacylglycerols
20	5.27 (m)	C$\underline{H}$-OCOR	Triacylglycerols
21(H)	5.35 (br)	C$\underline{H_2}$-CH = CH	All UFA

Note: The multiplicity of peaks: s, singlet; d, doublet; t, triplet; q, quartet; dd, double doublet; m, multiplet;

Table S2 Basic information of the experimental oil samples

Experiment contents	Types of edible Oil	Brands of edible oil	Numbers	Origins	Types of edible oil	Brands of edible oil	Numbers	Origins
Nutrient composition difference analysis and adulteration experiment	Camellia oil (Low temperature pressing)	DY	1	Bama, Guangxi	Olive oil	AN	7	Italy
		JH	2	Yongzhou, Hunan		AG	8	Greece
		QY	3	Hangzhou, Zhejiang		OG	9	Turkey
		RX	4	Yichun, Jiangxi		OV	10	Spain
		YL	5	Dabieshan, Anhui		DE	11	Spain
		DL	6	Shangrao, Jiangxi		QI	12	China
	Corn oil (Refine)	JY	13	Shenzhen, Guangzhou	-	-	-	-

Note: No. 1-12 edible oil samples were used for high-field NMR component analysis and low-field NMR relaxation time distribution analysis; No. 13 corn oil was used to blend camellia oil to make adulterated samples.
